# Influence of Polymeric Restorative Materials on the Stress Distribution in Posterior Fixed Partial Dentures: 3D Finite Element Analysis

**DOI:** 10.3390/polym13050758

**Published:** 2021-02-28

**Authors:** Larissa Mendes Campaner, Marcos Paulo Motta Silveira, Guilherme Schmitt de Andrade, Alexandre Luiz Souto Borges, Marco Antonio Bottino, Amanda Maria de Oliveira Dal Piva, Roberto Lo Giudice, Pietro Ausiello, João Paulo Mendes Tribst

**Affiliations:** 1 Institute of Science and Technology, São Paulo State University (Unesp), São José dos Campos, São Paulo 12220690, Brazil; larissa.m.campaner@unesp.br (L.M.C.); mttmarcos@gmail.com (M.P.M.S.); guisdandrade@hotmail.com (G.S.d.A.); alexanborges@gmail.com (A.L.S.B.); marco.bottino@unesp.br (M.A.B.); amodalpiva@gmail.com (A.M.d.O.D.P.); 2 Department of Clinical and Experimental Medicine, Messina University, AOU Policlinico “G.Martino”, Via Consolare Valeria, 98100 Messina, Italy; rlogiudice@unime.it; 3 School of Dentistry, University of Naples Federico II, 80131 Naples, Italy; pietausi@unina.it

**Keywords:** dental materials, finite element analysis, prosthodontics, biomechanics

## Abstract

Background: This study evaluated the effect of interim restorative materials (acrylic resin (AR), resin composite (RC) or polyetheretherketone (PEEK) for dental computer-aided design/computer-aided manufacturing (CAD/CAM)) on the stress distribution of a posterior three-unit fixed partial denture. Methods: The abutment teeth (first molar and first premolar) were modeled using the BioCAD protocol containing 1.5 mm of axial reduction and converging axial walls. A static structural analysis was performed in the computer-aided engineering software, and the Maximum Principal Stress criterion was used to analyze the prosthesis and the cement layers of both abutment teeth. The materials were considered isotropic, linearly elastic, homogeneous and with bonded contacts. An axial load (600 N) was applied to the occlusal surface of the second premolar. Results: Regardless of the restorative material, the region of the prosthetic connectors showed the highest tensile stress magnitude. The highest stress peak was observed with the use of RC (129 MPa) compared to PEEK and AR. For the cement layers, RC showed the lowest values in the occlusal region (7 MPa) and the highest values for the cervical margin (14 MPa) compared to PEEK (21 and 12 MPa) and AR (21 and 13 MPa). Conclusions: Different interim restorative materials for posterior fixed partial dentures present different biomechanical behavior. The use of resin composite can attenuate the stress magnitude on the cement layer, and the use of acrylic resin can attenuate the stress magnitude on the connector region.

## 1. Introduction

Interim dental restorations are used during prosthetic rehabilitation in order to provide function, phonetics and aesthetics to the patient during a short period of the treatment while the final restoration is not finished [1]. The interim restorations can be performed manually using acrylic resin (powder–liquid technique), resin composite or through computer-aided design/computer-aided manufacturing (CAD/CAM) technology [2]. Moreover, the creation of prosthetic manufacturing using the CAD/CAM process reaches a high level of clinical success. In addition, the digital process provides not only upgrades in the available dental materials but also a more precise final restoration [3]. The major advantage of CAD/CAM provisional materials is its industrial manufacture, reducing defect incorporation in the material bulk in comparison with the manual technique [4,5,6,7].

For many decades, the widely used material to manufacture the temporary restorations was acrylic resin polymethyl methacrylate (PMMA), commercially found as powder (polymer) and liquid (monomer). This material has some clinical disadvantages, such as low color stability and variable mechanical properties that depend on the handling conditions [8]. The PMMA CAD/CAM blocks have adequate Vickers hardness (20), flexural strength of 90 MPa and fracture toughness of 2.53 MN/m^3/2^ [9].

In the late-1990s, resin composites were introduced in dentistry, and currently, they are widely used to manufacture temporary restorations and final restorations. In contrast to acrylic resin, the resin composites contain fillers [8] in their composition, presenting superior strength and color stability [10,11]. In addition, they are easy to use, have better mechanical properties and can be used for different types of temporary restorations [12,13,14]. The CAD/CAM resin composite, with 61.9 to 70.5 filler content, has a high value of Vickers hardness (between 65 and 97.3) [15] and flexural strength between 9.7 and 14.7 MPa [15].

Another material that can be used to perform polymeric restorations is the polyetheretherketone (PEEK), a semicrystalline linear aromatic polyacrylic polymer [16]. PEEK is a rigid, grayish-colored material. It has excellent thermal stability [17], and its properties are not modified during the sterilization process [14]. It also presents an elastic modulus close to that of the human bone and dentin, in addition to being biocompatible with the oral tissues [18]. In dentistry, this material is widely used as a temporary abutment for implants during prosthetic rehabilitation [19]. However, it still presents some limitations regarding adhesion to the cement due to its low surface energy [20]. Since PEEK has a high mechanical performance, in some cases, it can be applied as an alternative biomaterial for dental prosthesis [21]. The use of PEEK to manufacture dental prosthesis is limited due to its grayish color and opacity; however, it can be used as a bilayer design in cases of totals crowns [20]. In addition, this material can be used as an alternative for metal alloys [21,22,23] and PMMA for CAD/CAM restorations [5,24]. Fixed partial denture (FDP) in PEEK, manufactured using the CAD/CAM technology, is more resistant when compared to pressed PEEK prostheses [25] and when compared to brittle materials [26]. In addition, the CAD/CAM PEEK has a high value of Vickers hardness (31.55 ± 2.67) [27], flexural strength of 26.7 ± 4.3 MPa [27] and fracture toughness between 1.4 and 0.8 MN.m^1/2^ [28].

To understand the behavior of interim restorations and the influence of restorative materials, an in silico comparison can be performed using the finite element analysis (FEA). FEA can assist in diagnosing mechanical problems by assessing the state of stress and strain of the materials and adhesive interfaces [29]; it can also evaluate the performance of materials with applications of specific geometries, resistance, stiffness or fatigue tests [30,31,32,33].

Therefore, the aim of this study was to evaluate the effect of interim restorative materials (acrylic resin (AR), resin composite (RC) or PEEK for CAD/CAM) on the stress distribution of a posterior three-unit FDPs. The null hypothesis was that there would be no difference in the mechanical behavior for the different materials.

## 2. Materials and Methods

For the present study, the three-dimensional geometries were modeled using Computer Aided Design software (Rhinoceros 5.0 McNeel North America, Seattle, WA, USA). A 3D model of partial right jaw from São Paulo State University database (UNESP–ICT São José dos Campos) was selected in stereolithography file and exported to the CAD software. The geometric model with the lower teeth (First Molar, Second Premolar and First Premolar) macrostructure was separated to be used as a volumetric model. For that, the command “reduce mesh”, available with a plugin in CAD software, was used with 50% of relevance, allowing more smooth structure with all normal face to be oriented in the same direction. The next step was the use of a reverse engineering plugin to reconstruct NURBS surfaces from mesh. Then, a 3D volumetric model of a first molar and first premolar was created based in the surface created by the curve network generated automatically. The central pontic was created at the same way, although without root [34]. The connector presented a rounded shape, and the area was 4.2 mm^2^ for both abutment teeth.

**Figure 1:**
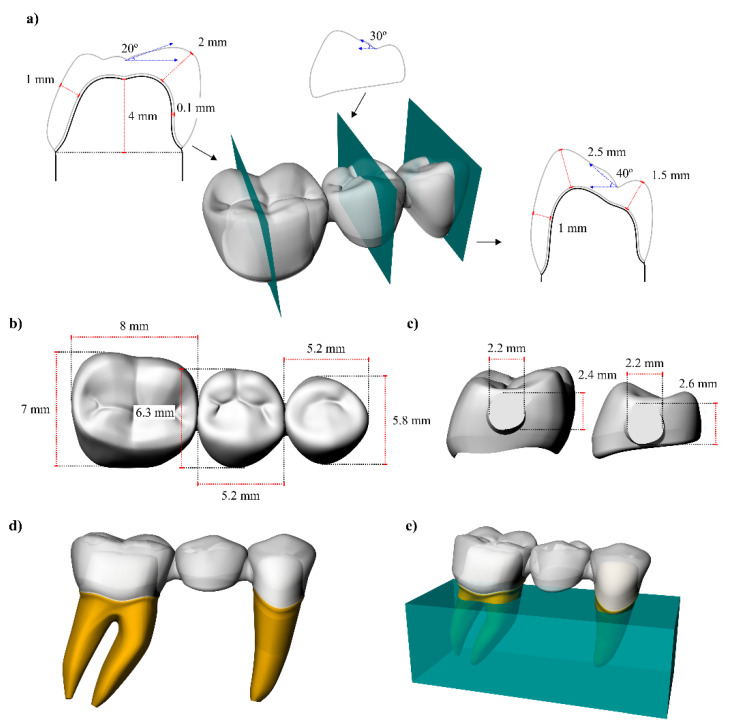
Three-dimensional modeling of fixed partial dentures in the computer-aided design software. (**a**) Section planes with each tooth profile and dimensions, (**b**) mesiodistal and buccolingual teeth dimension, (**c**) mesial and distal connectors height and width, (**d**) fixed dental prosthesis in position and (**e**) support model.

After the creation of the models, the crown preparations were performed. For both abutments, the preparation presented rounded corners, 6 degrees of axial wall reduction with 1.5 mm of thickness, 1.5 mm of occlusal reduction and a shoulder finishing line [32]. The cement layer was also modeled, with 100 μm thickness between the restoration’s intaglio surface and the teeth adhesive surface. For an isotropic substrate simulation, a polyurethane block (25 × 10 × 10 mm^3^) was created to embed the samples (Figure 1) [33].

Next, the models were exported in STEP format to the analysis software (ANSYS 19.2, ANSYS Inc., Canonsburg, PA, USA), where meshes were created using tetrahedral elements. The materials were considered isotropic, homogeneous and linearly elastic. The properties required for the mechanical analysis are summarized in Table 1 [34,35,36,37,38,39,40].

For the meshing, the convergence test was based on the number of nodes (350,628) and elements (185,842) obtained with 10% of relevance. The element used in the mesh division was the tetrahedral with 10 nodes (Tet-10). The mesh quality parameters were element quality defined as 0.72 ± 0.12, aspect ratio of 1.93 ± 0.84, average maximum corner angle of 96.43° and skewness average of 0.24 ± 0.15. The inflation option was defined as a smooth transition between the geometries. The rigid body behavior has been standardized as dimensionally reduced.

**Figure 2:**
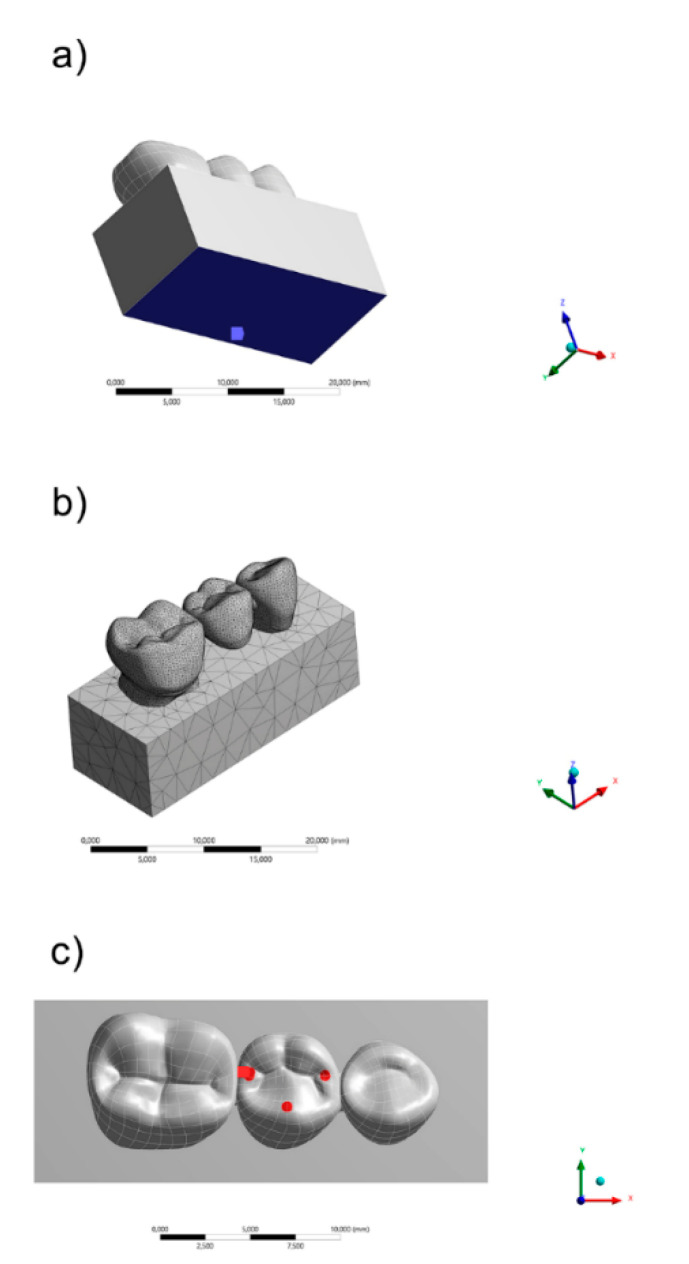
Finite element model showing (**a**) fixed support, (**b**) meshing division and (**c**) occlusal loading.

The fixed support was defined on the block’s bottom surface and the axial load of 600 N [30] was applied at the center of the pontic tooth (Figure 2). Values of maximum principal stress were evaluated through colorimetric graphs and stress peaks for each group through bar graphs [29]. The constitutive law in this study followed the Robert Hooke principle for linear elastic materials. The stress–strain relation was applied, assuming the general behavior of isotropic structures. The applied force in the determined area was calculated as well as the general strain after the change in the material’s dimension. For that, only the elastic modulus and Poisson ratio were necessary, since the bulk modulus and shear modulus were automatically calculated by the computer-aided engineering software.

## 3. Results

**Figure 3:**
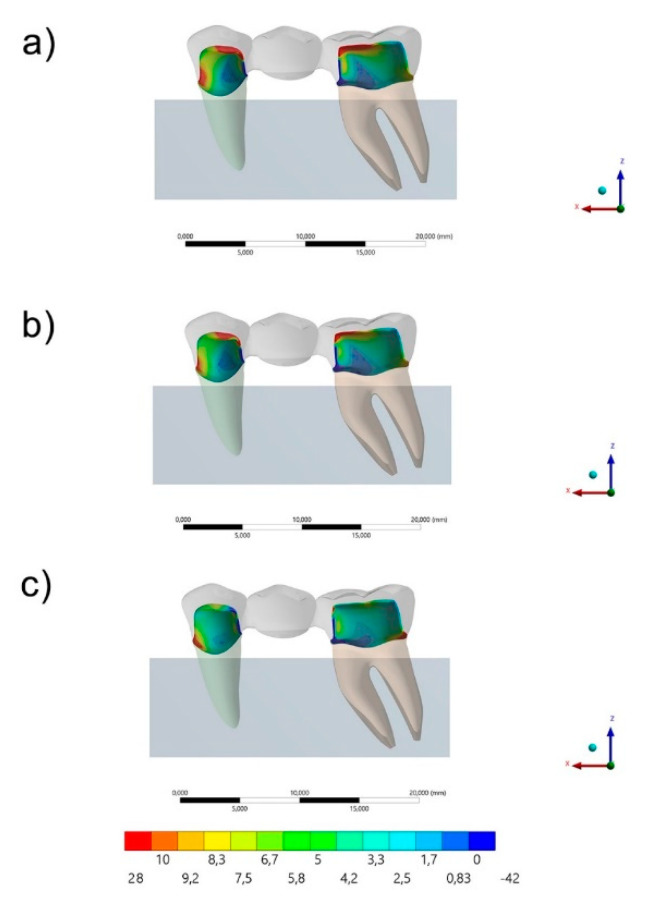
Sagittal view of the maximum principal stress maps (MPa) in the cement layer for each group: (**a**) acrylic resin (AR), (**b**) polyetheretherketone (PEEK) and (**c**) resin composite (RC).

**Figure 4:**
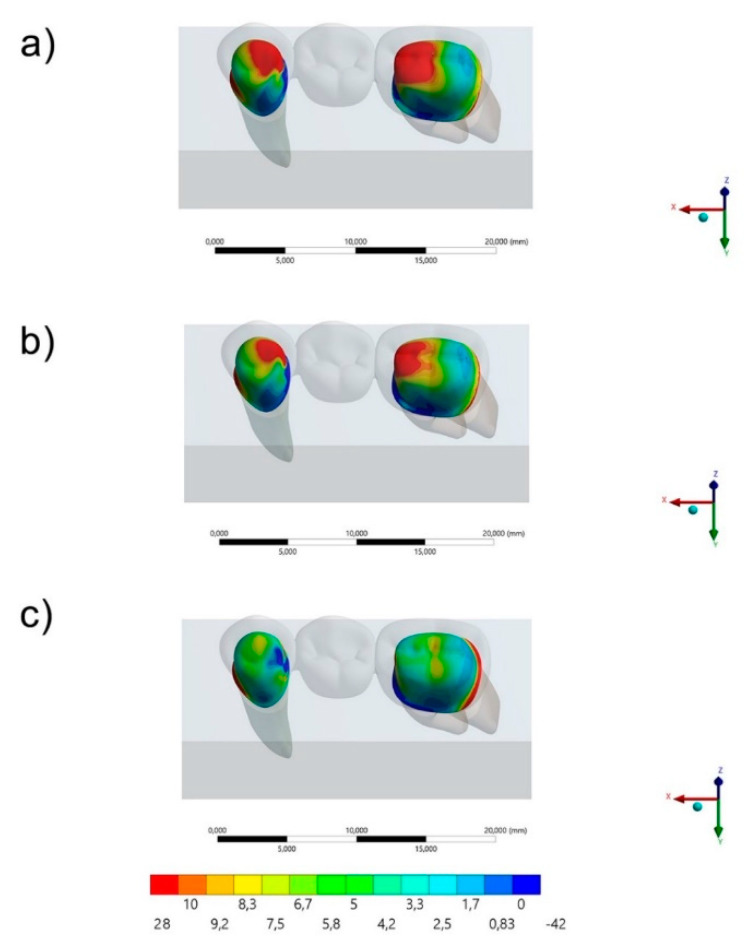
Occlusal view of the Maximum principal stress maps in the cement layer for each group: (**a**) AR, (**b**) PEEK and (**c**) RC.

**Figure 5:**
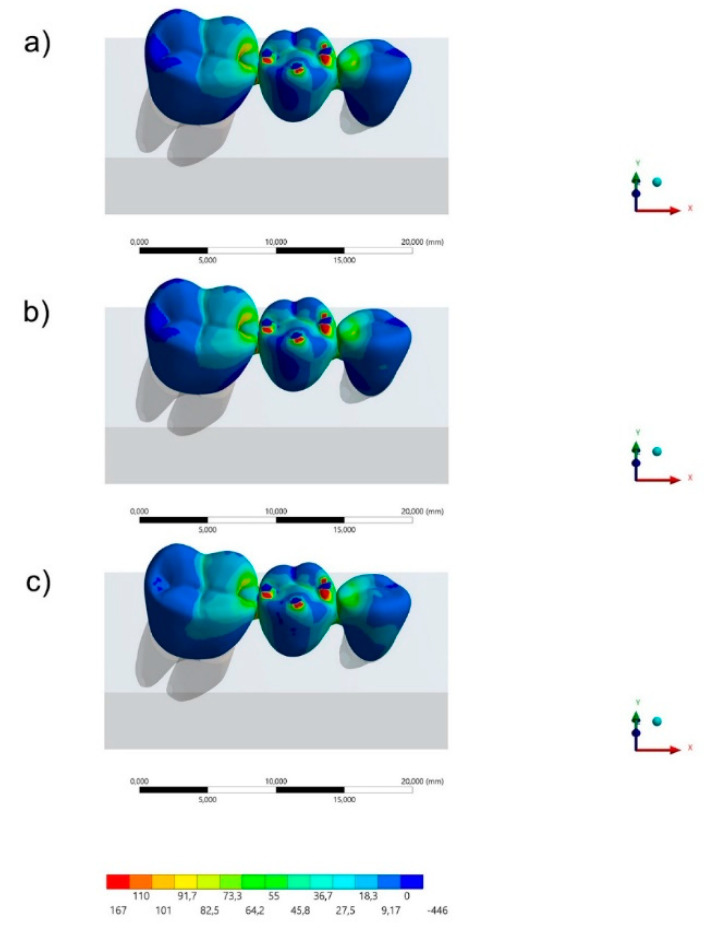
Occlusal view of the Maximum principal stress maps in the fixed partial denture (FDP) for each group: (**a**) AR, (**b**) PEEK and (**c**) RC.

**Figure 6:**
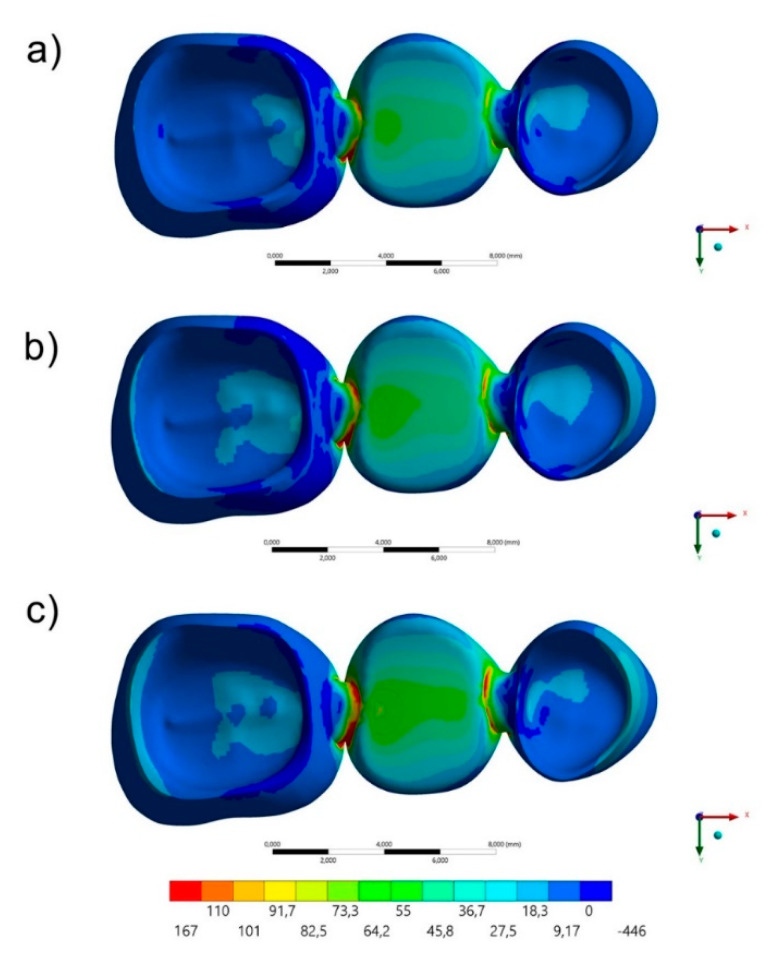
Bottom view of the Maximum principal stress maps in the FDP for each group: (**a**) AR, (**b**) PEEK and (**c**) RC.

The maximum principal stress distribution in the FDP and cement layers for all groups are displayed in Figure 3, Figure 4, Figure 5 and Figure 6. The stress peaks are summarized in Table 2.

Regarding the FDP’s behavior, it was possible to observe (Figure 3 and Figure 4) that the region of prosthetic connectors concentrated higher stress magnitude regardless the restorative material. The molar’s connector showed a higher stress concentration than the premolar’s connector, regardless the restorative material. However, the lower the elastic modulus used in the FDP, the lower the stress concentration in the connectors region. In the Figure 3, it is possible to see the stress concentration in the FDP from an occlusal view.

The stress maps for the cement layer are summarized in Figure 5 and Figure 6. In a section plane (Figure 6), the groups with acrylic resin (AR) and PEEK as restorative materials showed similar stress distribution pattern with higher stress concentration in the cervical region of the mesial face and occlusal of the distal face of the premolar and the occlusal of the mesial and cervical of the distal face of the first molar. For the resin composite (RC) group, the highest stress concentration occurred in the cervical region of the mesial face of the premolar and in the distal face of the first molar in the cervical region. In Figure 5, in an occlusal view, it is possible to observe that the higher the elastic modulus used in the FDP, the lower the stress concentration in the cement’s occlusal face.

## 4. Discussion

The objective of this study was to evaluate the influence of different interim restorative materials on the stress distribution of a fixed partial denture (FDP) using the finite element analysis. According to the obtained results, it was possible to observe that the null hypothesis was not accepted, since different restorative materials presented different mechanical behavior and stress magnitude. For the cement layer, the resin composite showed a promising behavior with the lowest stress concentration, followed by PEEK and then acrylic resin.

In the clinical practice, several failures modes in temporary restorations are reported, such as fractures and detachments. The regions that concentrate the highest stress in this in silico study corroborate with the most common failures of temporary restorations. In the literature, mechanical failures are reported in the FDP’s connectors, which can cause catastrophic fracture of the restoration and compromise the dental treatment [41]. Generally, these types of failures are associated with extensive rehabilitation or large edentulous spaces; however, there are no ideal restorative materials indicated to reduce this type of complication [42]. In this sense, the present study suggests that the acrylic resin should be used to reduce the stress magnitude in the connector region, thus preventing the possibility of a catastrophic failure.

A previous study [43] showed that 21% of the most common complications in FDPs involve their debonding. Based on this, the stress concentration in the cement layer justifies the reports of this failure type. Acrylic resin, resin composite and PEEK have shown, in previous studies [44], high bond strength values when the substrate receives an adequate surface treatment. In addition to debonding, the stress in the adhesive interface can also be associated with marginal infiltration [30,33,45], which can lead to postoperative sensitivity. Therefore, in this study, the acrylic resin showed the greatest chance of marginal infiltration over time.

The use of PEEK has been proposed in dentistry due to the positive results observed in medicine, showing indication to be applied as definitive material associated with ceramic veneering application [45,46]. In addition, it was reported [8] that PEEK is a rigid material with excellent thermal stability and resistance to wear without mechanical properties changing during the sterilization process. A previous study [21] aimed to compare FDPs in PEEK manufactured by different methods and their types of failures. The authors observed that three-unit FDPs manufactured from prepressed PEEK presented greater deformation and required higher loads (2354 N) to fracture when compared to the pressed ones in granular form (1738 N). Some studies report that PEEK is considered a suitable material for restorations in load-bearing occlusal areas [46,47,48,49,50]. However, due to the material elastic modulus, PEEK can be comparable with a temporary material [50]. The present study corroborated with that, showing that PEEK presented a mechanical behavior between RC and AR.

Resin composites are widely used to manufacture restorations because they present stability, excellent mechanical properties and a favorable aesthetic [51]. An in vitro study analyzed the stress relaxation behavior of acrylic resins and resin composites. The results showed significant differences in the stress relaxation behavior between both materials. Resin composites proved to be superior in their ability to maintain constant deformation without excessive dissipation of applied stress when compared to acrylic resins. The present study corroborated with this statement, showing a promising mechanical response for the cement layer when the resin composite was used in the FDP.

Regarding the treatment with FDPs, it was reported that the occlusal forces are transmitted to the surrounding structures through pontics, connectors and abutments and that the highest stress usually occurs in the connector region [52,53]. For this, four models were simulated using FEA, and the results showed that the maximum stresses were observed in the connectors. The present study showed a similar behavior between all interim restorative materials.

Temporary cements are used during the provisionalization phase. As a temporary material, they present favorable characteristics, such as high resistance to compression and, at the same time, an easy removal when it is necessary [53,54]. In this study, a visible stress concentration was observed in the cervical region by dispensing the load on the materials. This stress concentration can lead to cohesive failures in the cement layer. These flaws, in constant contact with oral fluids and other influencing factors, could increase the marginal infiltration that, in the long-term, can culminate in damages in the tooth structure and pulp [54,55,56]. Although the nanotechnology has brought improvements to the field of nanomedicine, providing improved materials that are able to mimic human body tissues to a certain point, there are still many challenges to overcome. Further effective studies on the adhesion control of microbial biofilm on the evaluated biomaterial surfaces could lead to possible solutions for preventing infections [56,57,58].

FEA is widely used in dental studies to analyze the biomechanical behavior of materials; however, there are also limitations to be considered that must be complemented with data from previous studies prior to a clinical decision [28,31,45,50,51]. The absence of pH simulation [51,57,58], biofilm, temperature and the use of isotropic materials are examples of limitations to be considered when evaluating the results. Biomimetic polymeric materials can also be applied in further studies considering the material mechanical properties [56,57]. FEA allows the assessment and study of areas under stress, applying a certain load in regions of possible failures that can be seen according to the geometry and mechanical behavior of the materials [32,59,60]. However, further fatigue lifetime should be evaluated for restorative materials, expressing its S–N curve to provide a more complete in vitro evaluation.

## 5. Conclusions

For CAD/CAM interim fixed partial dentures, the use of resin composite as a restorative material reduced the stress concentration in the cement layer, suggesting a beneficial response in the adhesive interface. The acrylic resin reduced the stress in the connector region, reducing the catastrophic failure possibility. PEEK presents an intermediate behavior between acrylic resin and resin composite mechanical response.

## Figures and Tables

**Table 1:** Mechanical properties of the materials/structures used in the current study.

Material/ Structure	Composition	Elastic Modulus (GPa) *	Poisson Ratio
Enamel	-	80	0.30
Dentin	-	18	0.23
Fixation cylinder	Polyurethane resin	3.6	0.30
Cement	Zinc oxide-based cement	1.35	0.30
Acrylic resin	Polymethyl methacrylate, diethyl phthalate, benzoyl peroxide, titanium dioxide	2.2	0.30
PEEK	100% Polyetheretherketone	3.0	0.30
Resin composite	UDMA, Bis-GMA, Bis-EMA, TEGDMA, Silica and fillers.	8.0	0.25

* Values obtained from the literature.

**Table 2:** Stress peaks (MPa) generated in the FDP and cement layers for each abutment tooth according to the evaluated restorative materials.

	Prostheses		Cement	
Material	Molar Connector	Premolar Connector	Molar Abutment	Premolar Abutment
AR	114.6	69.4	21.8	21.5
PEEK	123.1	78.5	21.5	15.5
RC	129.4	88.5	14.9	14.4

## Data Availability

The data presented in this study are available on request from the corresponding author.
